# Transcutaneous Drug Delivery Systems Based on Collagen/Polyurethane Composites Reinforced with Cellulose

**DOI:** 10.3390/polym13111845

**Published:** 2021-06-02

**Authors:** Narcis Anghel, Valentina Maria Dinu, Liliana Verestiuc, Irene Alexandra Spiridon

**Affiliations:** 1 “Petru Poni” Institute of Macromolecular Chemistry, Grigore Ghica–Voda 41, 700487 Iasi, Romania; vdinu@icmpp.ro; 2 Faculty of Medicine, Grigore T. Popa University of Medicine and Pharmacy, 9-13 Kogalniceanu Street, 700454 Iasi, Romania; liliana.verestiuc@bioinginerie.ro (L.V.); irenespiridon@yahoo.com (I.A.S.)

**Keywords:** collagen, polyurethane, cellulose, lignin, composites, drug release

## Abstract

Designing composites based on natural polymers has attracted attention for more than a decade due to the possibility to manufacture medical devices which are biocompatible with the human body. Herein, we present some biomaterials made up of collagen, polyurethane, and cellulose doped with lignin and lignin-metal complex, which served as transcutaneous drug delivery systems. Compared with base material, the compressive strength and the elastic modulus of biocomposites comprising lignin or lignin-metal complex were significantly enhanced; thus, the compressive strength increased from 61.37 to 186.5 kPa, while the elastic modulus increased from 0.828 to 1.928 MPa. The release of ketokonazole from the polymer matrix follows a Korsmeyer–Peppas type kinetics with a Fickian diffusion. All materials tested were shown to be active against pathogenic microorganisms. The mucoadhesiveness, bioadhesiveness, mechanical resistance, release kinetic, and antimicrobial activity make these biocomposites to be candidates as potential systems for controlled drug release.

## 1. Introduction

The beginning of the third millennium undoubtedly presents itself as a milestone in technological development. In a relatively short time, high-tech industries have shown an almost exponential development at the same time as the formulation of new concepts and theories. Thus, the reorientation is somewhat surprising, given the emergence of new intelligent materials for the use of natural products in different areas of activity that interfere with the biological field.

Natural polymers, given their intrinsic nature, are best suited for a wide variety of biomedical applications. Biodegradability and biocompatibility are key aspects for the formulation of drug delivery systems or for cell therapies such as wound dressing or their use as scaffolds for tissue engineering [1,2,3,4]. Herein, for the solubilization of cellulose, collagen, and polyurethane, in order to obtain transcutaneous transfer systems, a so-called green solvent was used which belonged to the category of ionic liquids, namely 1-(n-butyl)-3-methylimidazolium chloride [5]. The reason why the polymeric materials mentioned above were chosen for the design of topical composites for drug delivery devices was the fact that collagen ensures the bioadhesion of the material on the skin, polyurethane gives the necessary elasticity to the patches, and cellulose is a reinforcing material for the assurance of the mechanical strength.

Cellulose is one of the most widespread vegetable semicrystalline polymers, consisting of glucose units coupled by β-1,4-glucoside bonds [6]. Due to its hydrophilicity, it has a good absorption capacity, and for to its biocompatibility with the animal body, it is recommended for various biomedical or cosmetic applications [7,8,9].

At the same time, cellulose derivatives have proven to be versatile materials with multiple applications in a wide range of fields. Thus, cellulose derivatives have found their use in making seedling pots [10], surgical sutures [11,12], patches for topical applications [13,14,15], or for the design of biofriendly materials that have proven effective for heavy-metal retention [16,17,18,19,20]. The emergence of nanostructured biomaterials has paved the way for the design of cellulose-based composites for the controlled transport of drugs or other bioactive principles [21,22,23,24,25].

Collagen is probably the most abundant protein in the animal kingdom, forming the constitution of connective tissue, bones, tendons, and ligaments. For our studies, we considered type I collagen to be a component for composite transcutaneous transport systems due to its triple-helical structure that gives it a fibrillar morphology that has elasticity and mechanical strength. Cellulose/collagen composites have found successful applications in tissue engineering due to their biocompatibility with the human body [26,27].

Polyurethanes have proven to be interesting candidates for composite biomaterials used in vascular implants and grafts due to their biocompatibility with human body tissues [28,29,30,31,32]. In the same context, poly (ester-urethane)-containing phosphorylcholine segments have been shown to improve the hemocompatibility of blood-contacting medical devices [33]. On the other hand, the fine-tuning of the polyurethane structure allowed the design of materials with controlled biodegradability for long-term implant application [34,35]. Cellulose/polyurethane elastomers with good mechanical strength have proven to be high-performance and effective materials for wound treatment [36].

The incorporation of some drugs in the polymer matrix and their controlled release is an important way to control infections, inflammation, or tissue regeneration.

Lignin, probably the most widespread aromatic polymer of vegetable nature, has, among other things, important antioxidant properties, which make it attractive in terms of potential applications for the treatment of skin diseases [37,38,39,40]. At the same time, data from the literature stipulate the ability of lignin and ketoconazole to form complexes with some transition metals [41].

Thus, the biocomposites obtained were doped with a lignin or lignin-metal complex, which confers to these materials, the ability to control, through a large specific surface area and complexation capacity, the release of drugs with fungicidal properties such as ketoconazole.

Considering the interactions between the components of the matrix, lignin, and ketoconazole, the obtained materials were studied in terms of in vitro drug release, mucoadhesiveness and biological activity against pathogenic microorganisms. At the same time, morphology, mechanical properties, and interactions between system components were studied by scanning electron microscopy (SEM), compression tests, and Fourier transform infrared spectroscopy (FTIR).

## 2. Materials and Methods

### 2.1. Materials

Cellulose (cotton linters, ~20 micrometers, 240 Da), collagen hydrolysate, a polypeptide made by further hydrolysis of denatured collagen (molecular weight of 96 kDa), β-cyclodextrine, methylene diphenyl diisocyanate, and polycaprolactone (M_n_ ~ 2000) were purchased from Sigma-Aldrich and used without further purification. Ketokonazole was from Supelco, and organic lignin was extracted with acetic acid/phosphinic acid from birch wood [42].

### 2.2. Methods

#### 2.2.1. Polyurethane Synthesis

The polyurethanes used in this study were synthesized from polycaprolactone (PCL), methylene diphenyl diisocyanate (MDI), and a mixture of butane diol (BD) and beta-cyclodextrin (β–CD) at ratio of 9/1 (*w/w*), as chain extender in DMF solution. In brief, the PCL was dried in vacuum 1 mm Hg at 80 °C for 3 h. The reaction was carried out under stirring with MDI at the temperature of 80 °C for 1 h, and then the DMF solution mixture of BD and β–CD were added, and the mass reaction was kept under stirring at 60 °C for 6 h. The polyaddition reaction was stopped with a solution of 5 mL EtOH:DMF 1:1 (*v*/*v*) at the viscosity of ~7000 cP. Molar ratio between components was PCL/MDI/(BD/β–CD) of 3/4/1 [43].

#### 2.2.2. Preparation of Biomaterials

The reference material CCP (cellulose-collagen-polyurethane) was obtained by dissolution of cellulose (1 g), collagen (0.25 g), and polyurethane (0.25 g) in buthyl-3-methylimidazolium chloride (10 g) under stirring, at temperature of 100 °C for 8 h. Other biomaterials named CCPK (cellulose-collagen-polyurethane-ketoconazole), CCPOLK (cellulose-collagen-polyurethane-organosolv lignin-ketoconazole), CCPLMCK (cellulose-collagen-polyurethane-lignin metal complex-ketoconazole), CCPOL (cellulose-collagen-polyurethane-organosolv lignin), and CCPLMC (cellulose-collagen-polyurethane-lignin metal complex) were obtained by addition of 0.15 g lignin or lignin-ferrite hybrid (obtained in our laboratory by combustion of lignin with cobalt nitrate Co(NO_3_)_2_ 6H_2_O and ferric nitrate Fe(NO_3_)_3_ 9H_2_O at 500 °C) and 0.15 g ketoconazole to the cellulose–collagen–polyurethane matrix, by case. Biocomposites were obtained by casting in Petri dishes. After 48 h, the samples were washed with distilled water and dried.

### 2.3. Characterization

#### 2.3.1. FTIR Spectroscopy

FTIR spectroscopy was used to analyze the possible interactions materials’ components. In total, 64 scans of all samples were acquired using a Bruker, Vertex 70 (Billerica, MA, USA) ATR-FTIR spectrometer, equipped with ATR device (ZnSe crystal) with a 45 angle of incidence. The scan was recorded in a range from 4000 to 600 cm^−1^ and a spectral resolution of 2 cm^−1^.

#### 2.3.2. Scanning Electron Microscopy

SEM was used to analyze the cross-sections of material using a SEM (FEI QUANTA 200ESEM instrument) with an integrated EDX system, GENESIS XM2i EDAX with an SUTW detector. The samples were analyzed with a low-vacuum secondary electron detector at an accelerating voltage of 25.0 kV, at room temperature, and 0.050 Torr internal pressure. The experiment was performed in triplicate, at a magnification of 10,000×.

### 2.4. Bioadhesivity Test

An TA.XT plus^®^ analyzer from Stable Micro Systems (Godalming, UK) was used to evaluate the adhesion force (maximum detachment force) and total work of adhesion. The bioadhesion tests were performed on cellulose dialysis membrane, preboiled and cooled before testing, in a physiologically simulated environment of 37 °C, with a pH of 7.4 value (100 µL PBS added to each probe) and 250 rpm. The mucoadhesion tests were performed on porcine small intestine (bowel), in a physiologically simulated environment of 37 °C, with a pH of 7.4 value (100 µL PBS added to each probe) and 250 rpm.

### 2.5. Compression Test

The mechanical properties were investigated on EZ-LX/EZ-SX Series Shimadzu Testing Machine (Kyoto, Japan) in compression mode. All measurements were performed at room temperature, and the hydrogel samples were tested in swollen state after immersion in PBS. For compressive stress, the hydrogel samples were shaped as plate specimens with the thickness of 9 mm, width of 12 mm, and height of 2 mm. Before each measurement, an initial force of 0.1 N was applied to the hydrogel samples to ensure the complete contact between entire biomaterial surfaces and the parallel compression plates of the testing equipment. For each hydrogel sample, three measurements were registered at a cross-head speed of 1 mm min^−1^, and the values of Young modulus were presented as an average value ± SD. The force applied was kept constant for all samples at 100 N. The compressive stress (*σ*, kPa) was calculated as the normal force acting perpendicular to the area of the un-deformed specimen, whereas the strain (*ε*) was expressed as the ratio between the deformed and initial length of the hydrogel sample.

### 2.6. In Vitro Release Studies

The experiments were carried out in phosphate buffer saline (PBS) pH 7.2 at 37 ± 0.5 °C. Aliquotes of 0.5 mL of the supernatant were removed at different time intervals and diluted to a total volume of 3 mL, prior to examination. The amount taken was replaced with fresh PBS in order to maintain the sink conditions. The concentration of the released ketoconazole was analyzed spectrophotometrically at *λ_max_* value of 254 nm, at room temperature. The concentrations were calculated based on the calibration curves determined at the same wavelength. The drug-release kinetics was evaluated using the equation proposed by Korsmeyer and Peppas (Equation (1)) [44].

(1) $$\frac{M_{t}}{M_{\infty }}=k\times t^{n}$$

where *M_t_*/*M_∞_* represents the fraction of the drug released at time *t*; *M_t_* and *M_∞_* are the absolute cumulative amount of drug released at time t and the maximum amount released in the experimental conditions used, at the plateau of the release curves; k is a constant incorporating the characteristics of the macromolecular drug loaded system; and *n* is the release exponent, which is indicative of the release mechanism.

In the equation above, a value of *n* < 0.5 indicates a Fickian diffusion mechanism of the drug from the biomaterial sample, while a value 0.5 < *n* < 1 indicates a non-Fickian behavior. When *n* = 1, a case II transport mechanism is involved with zero order kinetics, while *n* > 1 indicates a special case II transport mechanism [45].

### 2.7. Antimicrobial Activity

Culture media were prepared from *Salmonella typhymurium*, *Escherichia coli* 25,922, and *Candida albicans* strains in peptone saline with a turbidity of 1° McFarland. A suspension of approximately 1500 UFC (colony-forming units/mL) was obtained by dilution. The surface of the tested materials, as well as of the control, was contaminated with 100 μL ATCC strain (*Salmonella typhymurium* 14,028, *Escherichia coli* 25,922, and *Candida albicans*). The inoculate was extracted using a sterile swab soaked in peptone saline and seeded on the surface of the specific environment (XLD, VRBG, and ALOA) after 24 h, and the plates were incubated at 37 ± 1 °C for 24 h. The colonies were counted, and the values were compared to the control.

## 3. Results and Discussions

### 3.1. FTIR Analysis

FTIR spectroscopy was used to analyze the possible interactions between material components.

**Figure 1:**
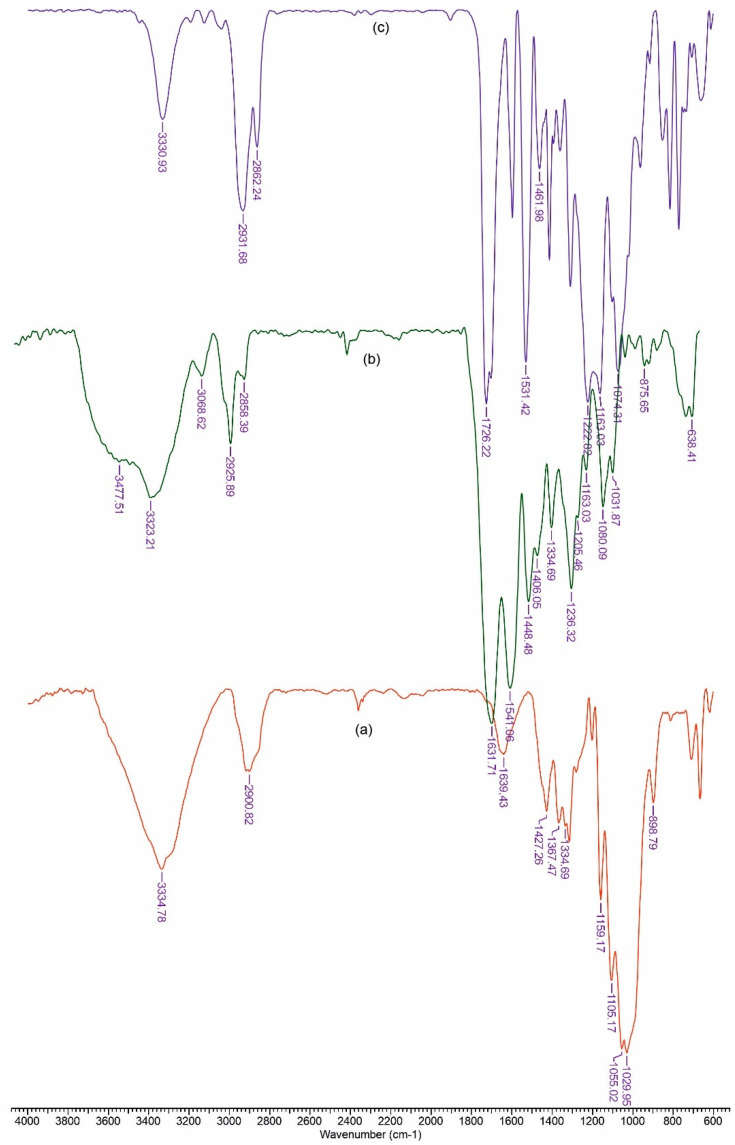
FTIR spectrum for cellulose (**a**), polyurethane (**b**) and collagen (**c**).

Figure 1 depicts the FTIR spectra for components of the reference film. The peaks observed in cellulose spectra (Figure 1a), in the range of 3660–2900 cm^−1^, are characteristic for the stretching vibration of O–H and C–H bonds in polysaccharides, while the broad peak at 3334 cm^−1^ is characteristic for the stretching vibration of the hydroxyl group in polysaccharides [46]. The band at 2900 cm^−1^ is attributed to the C–H stretching vibration of all hydrocarbon constituents in polysaccharides. Typical bands assigned to cellulose were observed in the region of 1630–900 cm^−1^. The peaks located at 1631 cm^−1^ correspond to the vibration of water molecules absorbed in cellulose [47]. The absorption bands at 1427, 1367, 1334, 1029, and 898 cm^−1^ belong to the stretching and bending vibrations of –CH_2_ and –CH, –OH and C–O bonds in cellulose [48,49].

Figure 1b shows the Fourier transform infrared spectrum recorded for polyurethane. The absorption band at 3323 cm^−1^ corresponds to NH stretching. The sharp peaks at 2858 and 2925 cm^−1^ are associated with –CH_2_ stretching, while other modes of –CH_2_ vibrations are identified by the bands at 1448, 1406, 1334, and 1236 cm^−1^. In addition, the absorption band at 1631 cm^−1^ is associated with a C=O group in polyurethane. The group of NH bend vibrations is identified by the band at 1631 cm^−1^ [50].

The spectra of collagen (Figure 1c) shows the amide A band, associated with N–H stretching, at 3330 cm^−1^. The amide bands were observed at 1631, 1541 and 1334 cm^−1^, respectively. Polypeptide backbone C–O stretching vibration was found in the range of 1600–1700 cm^−1^. C–N stretching vibrations were noted at 1222 cm^−1^ [51].

**Figure 2:**
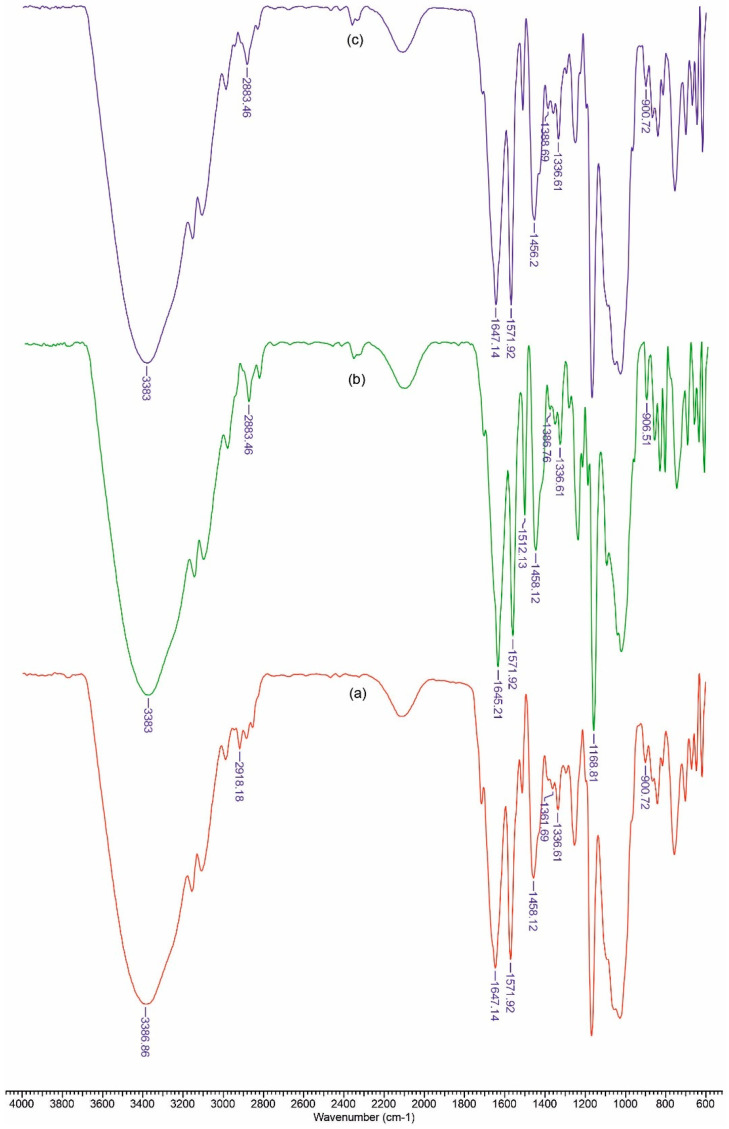
FTIR spectra for CCPK (**a**), CCPOLK (**b**) and CCPLMCK (**c**).

**Figure 3:**
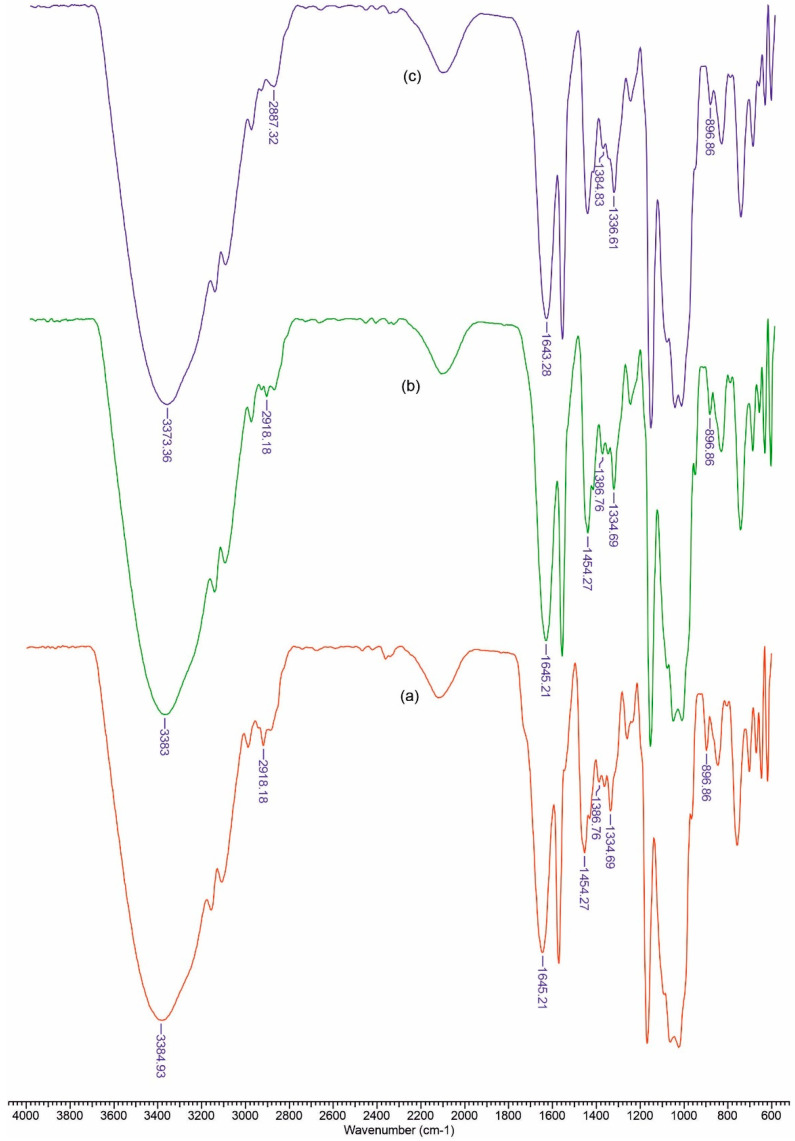
FTIR spectra for CCPLMCK(**a**), CCPOL (**b**) and CCP (**c**).

In Figure 2 and Figure 3 FTIR spectra are presented for the obtained materials. The spectra data show absorption bands characteristic for amino or carboxy groups that overlap with those of the vibration spectra in cellulose. The usefulness of these spectra lies in the fact that it allows the calculation of some structural parameters, as can be seen below.

Based on the recorded spectra, we can calculate a series of indices that reflect the degree of ordering and the total crystallinity, as well as the strength of the hydrogen bonds, for the studied materials.

The ratio between the heights of the bands at 1376 and 2902 cm^−1^ was proposed by Colomn and Carrillo [52] as the total crystalline index (TCI). The band at 1437 cm^−1^ is associated with the crystalline structure of cellulose, while the band at 899 cm^−1^ is assigned to the amorphous region in the cellulose. The ratio between the absorbance of the bands at 1437 and 899 cm^−1^ is used as a lateral order index (LOI). Considering the chain mobility and bond distance, the hydrogen bond intensity (HBI) of cellulose is closely related to the crystal system and the degree of intermolecular regularity—that is, crystallinity. The ratio of the absorbance bands at 3336 and 1336 cm^−1^ was used to study the cellulose sample’s HBI. The obtained results are displayed in Table 1. The TCI is proportional to the degree of crystallinity of cellulose, and LOI represents the ordered regions perpendicular to the chain direction in the cellulose.

The CCP samples exhibited the highest TCI and a low LOI, which implies the highest crystallinity degree and an increase in ordered regions perpendicular to the chain direction in cellulose. The data from Table 1 show that the CCPOLK material presents the highest LOI and a low value of TCI. It is possible that a lateral ordered cellulose structure was obtained in the cellulose-collagen-polyurethane matrix by the addition of organic lignin along with ketoconazole. At the same time, the HBI value for CCPOLK and CCPLMCK increased as compared to that of the matrix, which means that fewer available hydroxyl groups in the cellulose chain are able to interact by inter- and/or intra-molecular hydrogen bonding.

### 3.2. Mechanical Properties

**Figure 4:**
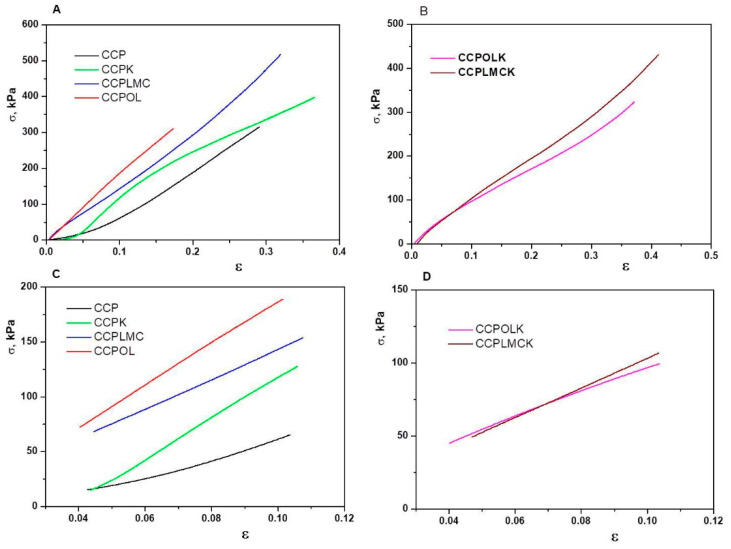
(**A**,**B**) Stress–strain curves obtained upon uniaxial compression onto swollen CCP-based hydrogels; (**C**,**D**) linear part of stress–strain curves from which the compressive Young’s modulus of all hydrogel samples was calculated.

In order to examine the effect of fillers on the mechanical strength of biomaterials, CCP-based hydrogels with different fillers including lignin (OL), lignin hybrid (LMC), and/or ketoconazole (K) were prepared. Typical stress–strain compression curves are revealed for all hydrogel samples (Figure 4), and the values of the compressive Young’s modulus and compressive strength of all hydrogels are depicted in Table 2. ANOVA (analysis of variance) test for all biocomposites was performed for a 5% confidence interval (alpha parameter was set to 0.05). *p*-value (0.001077) is less than the significance level (0.05); therefore, the null hypothesis (all materials have the same mechanical strength) is rejected. The Fisher parameter F (8.745) being larger than Fcrit (3.105) shows that there is a significant difference between the strength properties of the biomaterials in question, with CCPOL being a better candidate.

The CCP-based hydrogel without fillers exhibits weaker compressive mechanical strength with a value of the compressive Young’s modulus of 0.828 MPa and a compressive strength of 61.37 kPa (Figure 3A,C and Table 2). With the addition of fillers in the hydrogel-based materials, the mechanical strength of the hydrogel formulations increased gradually. When the lignin hybrid (LMC), ketoconazole (K), or lignin (OL) were incorporated into CCP based hydrogels, the compressive Young’s modulus was 1.63, 2.26, and 2.33 fold higher than those of CCP-based hydrogel without fillers (Table 2). The improvement of the compressive mechanical properties of the filler-loaded CCP hydrogels is well correlated with the HBI and LOI values which are higher for the filler-containing CCP hydrogels than for neat hydrogel (CCP without fillers). Similar behavior has been also reported for xanthan-based cryogels [53]. When second filler, i.e., ketoconazole (K), was incorporated within CCPLMC or CCPOL hydrogels, the values of the Young’s modulus and compression strength were diminished. This result could be associated with the high LOI values obtained for these hydrogel samples, which indicate more rigid hydrogels, but the energy/force put on it cannot be transferred away in time. The cationic octa (*γ*-chloroammoniumpropyl) silsesquioxane-chitosan-oxidized hydroxypropyl cellulose hydrogels [54] have been previously reported to present a comparable behavior with our CCP-based hydrogels.

### 3.3. Bio-/Muco-Adhesivity Properties

The biocomposites based on collagen/polyurethane/cellulose present a surface with adhesive properties. Adhesion of materials to the cutaneous tissue is an important property for product efficacy. In Table 3, the results are presented from bioadhesion and mucoadhesion tests on cellulose dialysis membrane and porcine skin, respectively.

The mucoadhesion test was performed in order to measure the ability of the films to adhere onto the porcine skin. The mucin present in the mucus surface layer of porcine skin is rich in cysteine (>10% of the amino acids) and therefore in the thiol groups which can lead the formation of with organic lignin; thus, CCPOLK has the greatest mucoadhesion force. The total work of the adhesion values is in good agreement with those of the adhesive forces.

### 3.4. In Vitro Release of Active Drug From Biocomposites

The ketoconazole release profiles from the investigated materials were studied (Figure 4) to evaluate the potential delivery applications.

**Figure 5:**
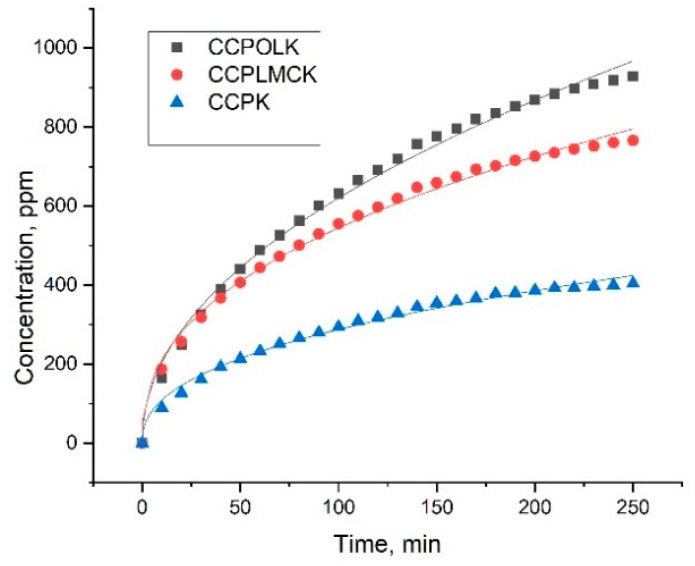
Release profiles of ketoconazole from the investigated samples.

Comparing results presented in Figure 5 and Table 4, it seems that the highest amount of the active substance was released from CCPOLK material, which is in a good agreement with the findings regarding mucoadhesive properties (Table 3). For all samples, the correlation coefficient (R^2^) was rather high (0.990) and describes the Korsmeyer–Peppas model. The transport constants (*k*) and transport exponents (*n*) were determined from the obtained data.

The *k* value was higher for the CCPLMCK sample as compared to the CCPOLK sample. The value of *n* was less than 0.5 for all samples, and this corresponds to a Fickian diffusion, suggesting that the release mechanisms of ketoconazole was related to the physical diffusion/drug dissolution interaction of electrostatic forces or hydrogen bonds [55].

### 3.5. Antimicrobial Activity

From Table 5, it is clear that all tested materials have biocidal activity. Note that CCPLMCK proves to be an active biomaterial against all three microorganisms, with an inhibition rate of 100%.

### 3.6. Material’s Morphology

**Figure 6:**
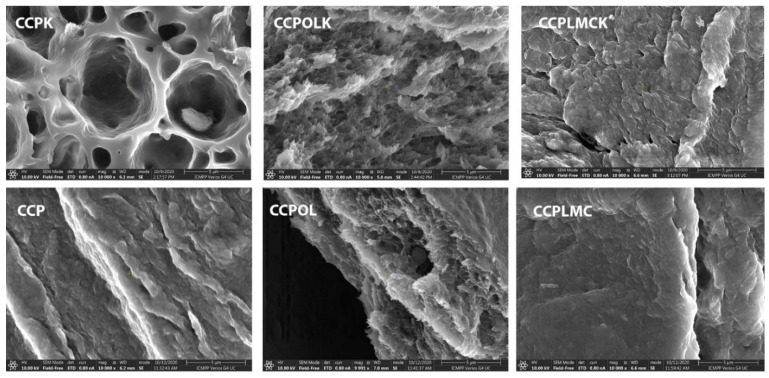
SEM images of the materials.

SEM images (Figure 6) can give important information about biocomposites morphology. When lignin or lignin-metal complex/ketoconazole were added into the matrix, its surface tends to become smoother. The presence of some microdomains suggested the development of hydrogen bonds network between matrix components and lignin/ketoconazole, in agreement with HBI value from Table 1.

## 4. Conclusions

Collagen/polyurethane/cellulose-based composite materials that function as transcutaneous drug delivery systems have been developed. Doping the polymer matrix with lignin or lignin-metal complexes induces an increase in mechanical strength and gives composites a controlled drug release character as well as biocidal activity. Compressive strength and the elastic modulus of biocomposites comprising lignin or lignin lignin-metal complex were significantly enhanced; thus, the compressive strength increased from 61.37 to 186.5 kPa, while the elastic modulus increased from 0.828 to 1.928 MPa. The improvement of biological properties is also confirmed by the increase in mucoadhesivity. In vitro release of ketoconazole from the biocomposites is described by the Korsmeyer–Peppas model with a Fickian diffusion. All materials tested were shown to be active against pathogenic microorganisms.

As a perspective, we have already considered the possibility of using these formulations in cosmetics, by introducing antioxidant substances in the base matrix.

## Figures and Tables

**Table 1:** Total crystalline index (TCI), lateral order index (LOI), and hydrogen bound intensity (HBI) values obtained from the FTIR spectra analysis of the biocomposites.

Sample	TCI (A_1376_/A_2902_)	HBI (A_3336_/A_1336_)	LOI (A_1437_/A_899_)
Cellulose	1.844	5.140	2.174
CCPK	1.625	4.114	2.849
CCPOLK	1.143	6.148	4.557
CCPLMCK	1.855	4.637	3.333
CCP	1.901	3.620	1.842
CCPOL	1.870	3.969	1.979
CCPLMC	1.436	3.985	1.836

CCPK—cellulose-collagen-polyurethane-ketoconazole; CCPOLK—cellulose-collagen-polyurethane-organosolv lignin-ketoconazole; CCPLMCK—cellulose-collagen-polyurethane-lignin metal complex-ketoconazole; CCP—cellulose-collagen-polyurethane; CCPOL—cellulose-collagen-polyurethane-organosolv lignin; and CCPLMC—cellulose-collagen-polyurethane-lignin metal complex.

**Table 2:** Values of the compressive Young’s modulus (E, MPa) and compressive strength of the swollen CCP-based hydrogels.

Sample Code	E, kPa	Compressive Strength, kPa			R^2^	Variance
CCP	828 ± 230	61.37 ± 1.21			0.989	52,900
CCPK	1877 ± 170	117.94 ± 2.07			0.999	28,900
CCPLMC	1357 ± 460	143.28 ± 3.42			0.999	211,600
CCPOL	1928 ± 280	186.58 ± 4.29			0.999	78,400
CCPLMCK	1014 ± 340	103.19 ± 2.11			0.999	115,600
CCPOLK	856 ± 140	96.78 ± 1.36			0.998	19,600
ANOVA (Analysis of Variance)						
Source of Variation	SS	df	MS	F	*p*-value	F crit
Between Groups	3,695,034	5	739,006.8	8.745643	0.001077	3.105875
Within Groups	1,014,000	12	84,500			

**Table 3:** Adhesive properties of the studied materials.

Sample	Bioadhesion Test		Muchoadhesion Test	
	Adhesion Force (N)	Total Work of Adhesion (N×s)	Adhesion Force (N)	Total Work of Adhesion (N×s)
CCPK	0.0588 ± 0.00264	0.0052 ± 0.0004	0.0555 ± 0.00346	0.00763 ± 0.0006
CCPOLK	0.04409 ± 0.00262	0.00183 ± 0.00046	0.0627 ± 0.0049	0.00746 ± 0.0007
CCPLMCK	0.0597 ± 0.00195	0.00576 ± 0.00041	0.0584 ± 0.0113	0.00736 ± 0.00056

**Table 4:** Kinetic parameters of the drug release from investigated samples.

Samples	*n*	*k*, min^−*n*^	R^2^
CCPK	0.424	40.54	0.990
CCPOLK	0.484	66.38	0.994
CCPLMCK	0.413	80.99	0.995

*n* = release exponent, *k* = release rate constant, R^2^ = correlation coefficient.

**Table 5:** Antimicrobial activity for tested biocomposites.

Sample	ATCC 25,922 *Escherichia coli*			*Candida albicans* 90,028			*Staphylococcus aureus* 25,923		
	Initial Number of Colonies	24 h	Inhibition, %	Initial Number of Colonies	24 h	Inhibition, %	Initaial Number of Colonies	24 h	Inhibition, %
Reference	287	287	0	255	255	0	293	293	0
CCP	287	3	99	255	183	28	293	11	96
CCPK	287	0	100	255	23	91	293	6	98
CCPOL	287	4	99	255	14	95	293	127	57
CCPOLK	287	0	100	255	5	98	293	93	68
CCPLMC	287	0	100	255	186	27	293	3	99
CCPLMCK	287	0	100	255	0	100	293	0	100
